# Risk Assessment of Methicillin- and Vancomycin-Resistant *Staphylococcus aureus* in Raw and Cooked Chicken Meat

**DOI:** 10.3390/foods15203620

**Published:** 2026-10-12

**Authors:** Rana Fahmi Sabala, Elham Elsayed Abo-Almagd, Amira Ibrahim Zakaria, Mahmoud El-Maghrabey, Mohammed A. S. Abourehab, Amany M. Shosha

**Affiliations:** 1 Department of Food Hygiene, Safety and Technology, Faculty of Veterinary Medicine, Mansoura University, Mansoura 35516, Egypt; 2 Faculty of Health Science Technology, Mansoura National University, Gamasa 7731168, Egypt; 3 Oncology Center, Faculty of Medicine, Mansoura University, Mansoura 35516, Egypt; 4 Department of Pharmaceutical Chemistry, Faculty of Pharmacy, University of Tabuk, Tabuk 71491, Saudi Arabia; 5 Pharmaceutical Science Department, College of Pharmacy, Umm Al-Qura University, Makkah 21955, Saudi Arabia; 6 Department of Bacteriology, Mycology and Immunology, Faculty of Veterinary Medicine, Mansoura University, Mansoura 35516, Egypt

**Keywords:** Raw and RTE chicken, antibiotic resistance, foodborne pathogens, MRSA, biofilm, quantitative risk assessment, Egypt

## Abstract

(1) Background: Methicillin-resistant *Staphylococcus aureus* (MRSA) has emerged as a critical foodborne pathogen, posing a significant threat to public health due to its multidrug resistance. In this study, we investigated the prevalence, antibiotic resistance, and virulence factors of *Staphylococcus aureus* (*S. aureus*) in raw and cooked chicken meat. (2) Methods: In total, 200 raw and cooked chicken meat samples were analyzed for *S. aureus* using phenotypic and molecular methods. Isolates were screened for antimicrobial susceptibility, enterotoxins, and biofilm formation, with the statistical analysis among the results. (3) Results: From 200 samples, 26% were contaminated with *S. aureus*, with mean counts reaching 2.08 × 10^4^ CFU/g in raw and 7.37 × 10^2^ CFU/g in cooked samples. Furthermore, 41.67% of the 96 *S. aureus* isolates were identified as MRSA, with 5.2% exhibiting vancomycin resistance. A high prevalence of multidrug resistance was observed, particularly against erythromycin, oxacillin, and ampicillin. In addition, the enterotoxin-encoding genes *sea* and *seb* were recovered from 47.92% and 28.13% of the isolates, respectively. Furthermore, 83.33% of the isolates demonstrated biofilm-forming capabilities, a factor associated with increased antibiotic resistance. Quantitative risk assessment revealed a non-negligible probability of illness associated with the consumption of contaminated chicken meat, highlighting the need for stringent food safety measures to mitigate this public health risk. (4) Conclusions: The high prevalence of MDR-MRSA in retail chicken is considered to have potential public health implications, suggesting the need for antibiotic stewardship in the poultry industry.

## 1. Introduction

*Staphylococcus aureus* (*S. aureus*) is one of the significant invisible threats lurking in our food chain, which is a critical foodborne pathogen leading to many outbreaks worldwide [1,2,3]. *S. aureus* is a uniquely resilient foodborne pathogen due to its ability to produce heat-stable enterotoxins and its increasing role as a reservoir of AMR genes in the poultry chain. *S. aureus* is a facultative anaerobic gram-positive bacterium widely spread in the environment and can survive different temperatures, humidity, and pH. It can be found in the environment, including air, dust, sewage, and water, besides humans and animals. Improper handling, food preparation errors, improper storage, and cross-contamination are the main causes of foodborne staphylococcal infection [4,5].

Food of animal origin, including chicken meat and its products, is considered an unwitting partner in foodborne pathogen dissemination, frequently serving as a reservoir for both bacterial contamination and antimicrobial resistance [6]. Several studies have highlighted the role of *S. aureus* in foodborne outbreaks associated with chicken meat consumption [2]. Staphylococcal food intoxication is an acute illness indicated by the onset of nausea, vomiting, diarrhea, and abdominal pain within 3–12 h after the consumption of food contaminated by *S. aureus* toxins [7]. The intoxication severity depends on the host susceptibility and the quantity of enterotoxin ingested. Hospitalization due to staphylococcal foodborne illness is uncommon and typically involves vulnerable individuals, such as infants, the elderly, or those with underlying health conditions; the ingestion of even small amounts, in the range of 20–100 ng, of preformed enterotoxins can be sufficient to induce illness [7].

Globally, *S. aureus* is responsible for an estimated 30.2% of foodborne illnesses, with higher rates in meat, cereals, and dairy and lower rates in vegetables and confectionery [2]. In the European Union, comprehensive surveillance data underscores the persistent burden of staphylococcal contamination. According to the European Food Safety Authority (EFSA), 5550 foodborne outbreaks were recorded in 2009, resulting in approximately 49,000 cases and 46 fatalities [8]. Of these, 293 outbreaks were attributed specifically to *Staphylococcus* spp. In North Africa and the Middle East, recent surveillance indicates a prevalence rate in retail poultry ranging from 15% to 35%, highlighting a significant regional burden [9].

Adding to concerns about food safety challenges after the COVID-19 pandemic is the growing presence of Methicillin-resistant *S. aureus* (MRSA) in food. This antibiotic-resistant species of *S. aureus* poses a significant public health threat, causing infections that are more challenging and expensive to treat. The MRSA epidemiology in food-producing animals has gained importance in recent years. This concerning trend can be traced back to the intensifying query for high-quality food and enhanced food safety. To face this increasing demand, the animal production, treating, processing, and distribution sectors have undergone substantial intensification.

Given the potential food safety implications of *S. aureus* contamination, establishing a clear regulatory framework is essential. International organizations such as the Codex Alimentarius Commission (FAO/WHO) and European Commission regulations (e.g., Commission Regulation (EC) No 2073/2005 on microbiological criteria for foodstuffs) establish strict microbiological limits for bacterial pathogens in ready-to-eat and raw foods [10,11]. In Egypt, microbiological standards and guidelines issued by the Egyptian Organization for Standardization and Quality (EOS) govern the acceptable limits of bacterial counts in meat products [12]. Understanding these regulatory requirements provides the practical and public health context necessary to evaluate the significance of the hazards investigated in this study.

Given the potential consequences of *S. aureus* contamination, a thorough risk assessment of *S. aureus* in food becomes a crucial line of defense. This involves identifying vulnerabilities in the food chain, from farm to fork, and implementing effective control measures to minimize their presence. This study was conducted to determine the prevalence of *S. aureus* isolates in raw and cooked chicken meat retailed in Mansoura City, Egypt. Furthermore, the research investigated the antibiotic susceptibility patterns of these isolates and evaluated their capacity for biofilm formation. In addition, the study assesses the potential risk associated with the consumption of such products contaminated by such microbes.

## 2. Materials and Methods

### 2.1. Study Design and Sample Collection

A total of 200 samples of raw and cooked chicken meat (100 each) were collected from November 2022 to April 2023 from various butchers and food vendors in Mansoura City, Egypt. Each sample was neatly wrapped in a plastic bag, labeled with the date and location, and promptly transported in an icebox to the laboratory of the Food Hygiene, Safety, and Technology Department in the Veterinary Medicine Faculty at Mansoura University, Egypt, for the isolation and detection of *S. aureus* contamination.

### 2.2. Isolation and Identification of S. aureus

A 25 g portion of each sample was aseptically transferred to 225 mL of Butterfield’s phosphate buffer and homogenized for 2 min. Subsequently, serial 10-fold dilutions were prepared in sterilized saline. From each dilution, 0.2 mL was spread-plated onto Baird–Parker agar supplemented with egg-yolk tellurite (Oxoid, Hampshire, UK) and incubated at 35 °C for 48 h. Characteristic morphology colonies (black with a clearing zone surrounded by the opaque medium) were taken. Up to five typical colonies from each plate were randomly picked and exposed to further identification by biochemical tests, including coagulase production, catalase activity, detection of hemolysis, and mannitol fermentation test. Only coagulase-positive *S. aureus* was considered in the final count, and the count was expressed as CFU/g of the original chicken meat sample. The presumptive colonies were then taken for further chemical and molecular identification.

### 2.3. Molecular Detection of S. aureus and Enterotoxins

All the suspected colonies of *S. aureus* were screened for the *nuc* gene, for the potential for the rapid diagnosis of *S. aureus* infections [13]. From 52 *S. aureus*-positive samples, a total of 96 distinct colonies were isolated and retained for further analysis to capture the phenotypic and genotypic diversity of the *S. aureus* population, and characterizations reflect isolate-level frequencies rather than direct sample-level generalizations. The confirmed isolates as *S. aureus* had been screened using singleplex PCR amplification for the enterotoxin-encoding genes (*sea* and *seb*) [14,15]. The amplification was done using DreamTaq^TM^ Green PCR Master Mix (2X), Cat. (Thermo Scientific, Waltham, MA, USA), where 35 cycles of denaturation at 94 °C/30 s, annealing at 57 °C for the screened genes for 30 s, and extension at 72 °C/30 s following an initial denaturation at 94 °C/3 min with a final extension at 72 °C/10 min, using the MiniPCR TM Mini16 Thermal Cycler (Amplyus, Cambridge, MA, USA). The electrophoresis was done in a 1.5% agarose gel using a blueGel detection and visualization unit (Amplyus, Cambridge, MA, USA). The primers used are shown in Table 1.

### 2.4. Antibiotic Susceptibility Testing

The antimicrobial susceptibility of the molecularly confirmed *S. aureus* isolates was carried out according to Clinical and Laboratory Standards Institute guidelines [16] using the Kirby–Bauer disc diffusion method using Mueller–Hinton agar (MH; CM0337; Oxoid Ltd., Basingstoke, UK) and antimicrobial susceptibility discs (Oxoid, Hampshire, UK). Briefly, bacterial suspensions were prepared in sterile saline and adjusted to a 0.5 McFarland turbidity standard. The inoculated plates were then incubated at 35 °C for 16–18 h before measuring the zones of inhibition. The antibiotics included ampicillin (AMP; 10 μg), oxacillin (OXA; 5 μg), ofloxacin (OFL; 5 μg), tetracycline (TET; 30 μg), chloramphenicol (C; 30 μg), gentamicin (GM; 10 μg), erythromycin (E; 15 μg), and trimethoprim/sulfamethoxazole (Co-trimoxazole) (Co-T; 1.25/23.75 µg). The susceptibility against vancomycin (Sigma, Aldrich, St. Louis, MO, USA) was tested by the broth microdilution method according to the CLSI [16,17], using ranges of concentrations of 0.5–32 μg/mL. Results were interpreted as susceptible, intermediate, or resistant according to the Clinical and Laboratory Standards Institute guidelines.

Isolates were categorized according to the standardized international terminology for antibiotic resistance [18]. Non-susceptibility was defined as any isolate testing either intermediate or resistant to at least one agent within a specific antimicrobial category. Following these criteria, multidrug-resistant (MDR) was defined as non-susceptibility to >1 agent in three antimicrobial categories. Extensively drug-resistant (XDR) was defined as non-susceptibility to >1 agent in all but >2 categories, while pan-drug-resistant (PDR) indicated non-susceptibility to all agents across all categories.

### 2.5. Molecular Analysis of the Antibiotic Resistance mecA and vanA Genes

The *S. aureus* isolates that showed resistance to oxacillin were screened for *mecA* gene [19]. In addition, the resistant *S. aureus* isolates against vancomycin with a MIC ≥ 16 μg/mL were screened for the *vanA* gene [20]. The amplification was done using DreamTaq^TM^ Green PCR Master Mix (2X), Cat. (Thermo Scientific, Waltham, MA, USA), where 35 cycles of denaturation at 94 °C/30 s, annealing at 57 °C for *mecA* and 54 °C for *vanA* gene for 30 s, and extension at 72 °C/30 s following an initial denaturation at 94 °C/3 min with a final extension at 72 °C/10 min, using MiniPCR^TM^ Mini16 Thermal Cycler (Amplyus, Cambridge, MA, USA). The electrophoresis was done in a 1.5% agarose gel using a blueGel detection and visualization unit (Amplyus, Cambridge, MA, USA). The primers used are shown in Table 1.

**Table 1:** Primers used in the current study for the molecular characterization of *S. aureus* isolated from raw and cooked chicken meat.

Primer	The Gene	The Sequence	Annealing Temperatures	Size bp	Reference
nuc F nuc R	*nuc* gene	5′-GCG ATT GAT GGT GAT ACG GTT-3′ 5′-AGC CAA GCC TTG ACG AAC TAA AGC-3′	57 °C	280	[13]
mecA F mecA R	*mecA* gene	5′-ACT GCT ATC CAC CCT CAA AC-3′ 5′-CTG GTG AAG TTG TAA TCT GG-3′	57 °C	163	[19]
vanA F vanA R	*vanA* gene	5′-GGGAAAACGACAATTGC-3 5-GTACAATGCGGCCGTTA-3′	54 °C	732	[20]
sea F sea R	*sea* gene	5′-TAA GGA GGT GGT GCC TAT GG-3 5′-CAT CGA AAC CAG CCA AAG TT-3	57 °C	180	Cremonesi [14]
seb F seb R	*seb* gene	5′-TCG CAT CAA ACT GAC AAA CG-3 5′-GCA GGT ACT CTA TAA GTG CC-3	57 °C	478	[15]

### 2.6. Biofilm Formation Assay In Vitro

The qualitative assessment of biofilm formation by *S. aureus* isolates was conducted using Christensen’s test tube method [21]. Each *S. aureus* strain was cultured in tryptone soya broth supplemented with 1% glucose, with an uninoculated broth serving as a negative control. Following incubation at 37 °C for 24 h, the broth was removed, and the tubes were stained with 1% crystal violet for 30 min. Non-adherent dye was then removed by washing each tube three times with sterile distilled water. Isolates were visually assessed as negative, weak, moderate, or strong according to the intensity and extent of the violet film adhering to the tube walls and bottom. All biofilm formation assays were performed in duplicate.

### 2.7. Quantitative Risk Assessment of Chicken Meat by S. aureus

The hazard characterization of *S. aureus* can be calculated using the dose-response model using the following equation:

P = 1 − exp (−r × D)

where P is the probability of illness, r is the probability of *S. aureus* single cells causing foodborne illness (*r* = 1.48 × 10^−8^) [22], and D is the dose of *S. aureus* cells ingested (CFU) in the current study. The ingested dose (*D*) was calculated using the formula *D* = *C* × *V*, where *C* represents the mean concentration of *S. aureus* detected in the chicken meat samples (CFU/g) and *V* represents the assumed standard serving size of chicken meat (100 g) based on regional Egyptian dietary consumption patterns. This deterministic-exposure framework integrates prevalence and mean concentration levels to estimate the relative probability of foodborne staphylococcal illness.

### 2.8. Statistical Analysis

Quantitative data for *S. aureus* counts are expressed as mean ± standard error (SE). To evaluate the distribution of *S. aureus* isolates across different sample types, as well as variations in virulence gene prevalence and antibiotic resistance rates, the chi-square (χ^2^) test was employed. The relationship between biofilm-forming capacity and multidrug resistance profiles was assessed using the nonparametric Spearman correlation coefficient (r_s_ = 0.84, *p* < 0.0001). All statistical analyses were performed using GraphPad Prism version 9.1.2 (GraphPad Software Inc., San Diego, CA, USA). For all tests, a *p*-value < 0.05 was considered to indicate statistical significance.

## 3. Results and Discussion

### 3.1. Prevalence of S. aureus in Chicken Meat Samples

The prevalence of *S. aureus* detected in the current study was 26% (52/200 raw and cooked chicken meat samples), divided into 32% (32/100) in raw and 20% (20/100) in cooked samples. In Egypt, *S. aureus* had been recovered from the poultry industry in high prevalence [23], while a lower prevalence was recovered only from 3% of the samples in another study [24]. For retail chicken meat, globally, the variable *S. aureus* prevalence, such as 23% and 41% in the US [25,26]; 41.8%, 53.8%, 54.9%, and 67.6% in North Dakota [27,28,29,30]; and 89.5% in the Republic of Serbia [31], has been recorded.

*S. aureus* was detected by mean counts of 2.08 × 10^4^ and 7.37 × 10^2^ CFU/g in raw and cooked chicken meat samples tested in this study, respectively. The *S. aureus* counts in 81% (26/32) of the *S. aureus*-positive raw chicken meat samples exceeded the permissible level according to the national and international guidelines instituted for assessing the microbiological safety of foods, as the threshold for an unsatisfactory level of *S. aureus* is ≥10^4^ CFU/g [32,33]. However, *S. aureus* counts in cooked chicken meat were within the permissible level, ranging from 1 × 10^2^ to 1.6 × 10^3^ CFU/g. Similar to the current study, unacceptable levels of *S. aureus* have been recorded in previous studies in Egypt and globally, reaching mean counts of 2.31 × 10^3^ and 1.4 × 10^8^ CFU/g [34,35]. The variable counts and contamination levels of food of animal origin by *S. aureus* organisms depend on the educational levels of food workers and the level of hygienic measures in the food chain. Therefore, strict hygienic measures should be taken by food workers using gloves, head covers, masks, and aprons during food handling and processing for consumers.

### 3.2. Prevalence of Nuclease (nuc) and Enterotoxin Genes Among the Coagulase-Positive S. aureus Isolates

**Figure 1:**
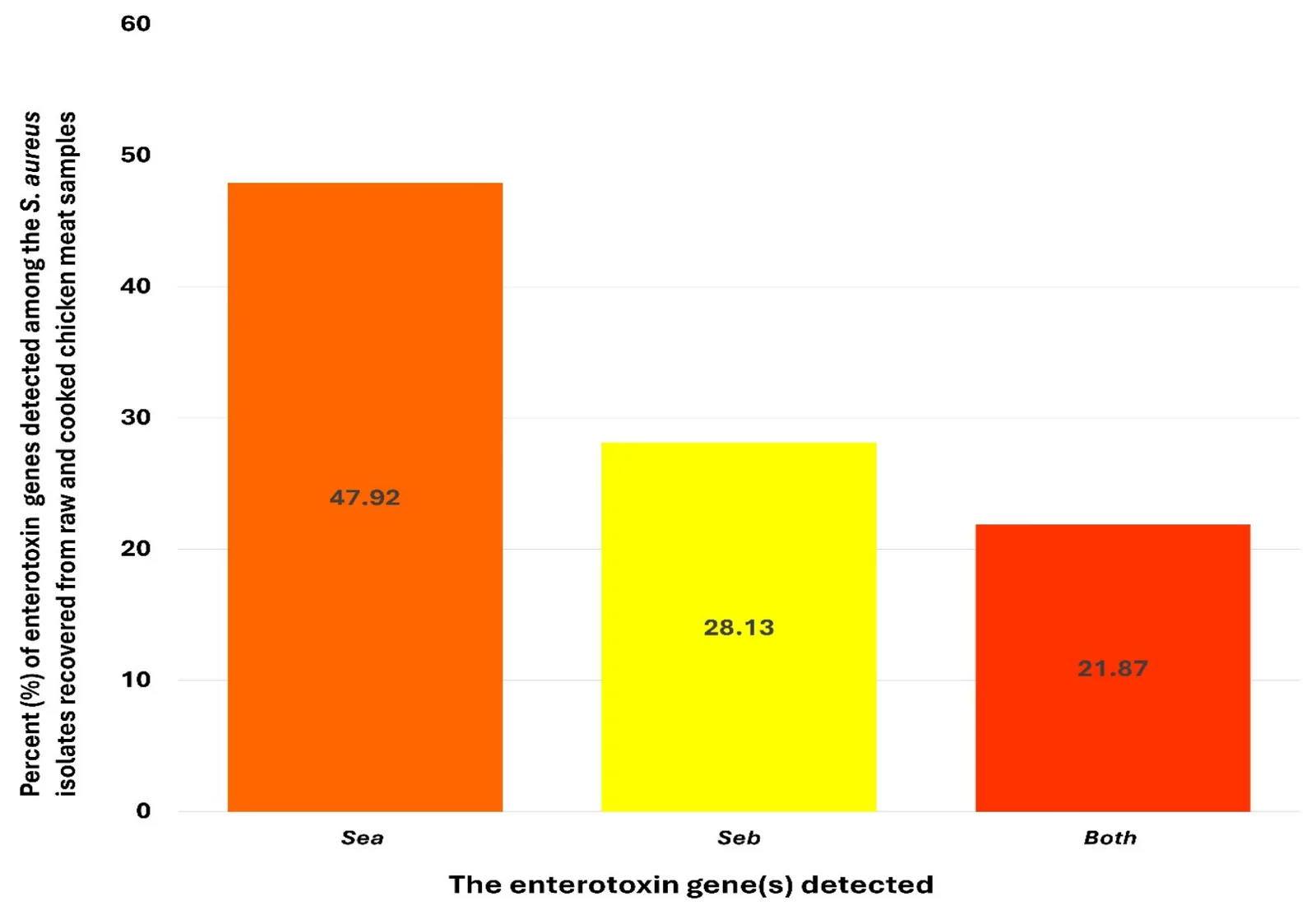
Prevalence of enterotoxin detected among the *S. aureus* isolates recovered from raw and cooked chicken meat samples. The screened genes in the current study were *sea* and *seb* genes among the recovered isolates (n = 96).

In the current study, a total of 96 coagulase-positive *S. aureus* isolates were recovered from the initial 32 raw and 20 cooked positive samples and confirmed by molecular amplification of the *nuc* gene, which served as a reliable molecular confirmation marker for *S. aureus* [36]. Concerning the enterotoxins (SEs) screening, the *sea* gene was recovered from 47.92% (46/96) of the *S. aureus* isolates, while the *seb* gene was detected in 28.13% (27/96), with 21 isolates carrying both genes (21.9%) (Figure 1). Overall, the vast majority of the isolates harbored one or more of the tested enterotoxin markers, with only two isolates lacking both tested enterotoxin genes. The detection of *sea* and *seb* genes among the *S. aureus* isolates highlights a significant genetic potential for enterotoxin production in retail poultry. However, because molecular methods identify genetic markers rather than active protein expression, these findings do not confirm the presence of biologically active enterotoxins or their survival following thermal processing. Gene screening provides valuable baseline surveillance data regarding enterotoxigenic potential. The virulence result in this study was in accordance with previous studies that tested the characteristics of *S. aureus* isolated from poultry and proved that all the isolates carried at least one enterotoxin gene, with the most common being *sea*. It is known that the *sea* and *seb* genes are related to contamination created by humans [37,38]. Although *sea* and *seb* have been reported in strains associated with human contamination, their detection alone does not establish a human origin of the isolates. Therefore, contamination may occur at various stages of production, handling, or preparation; the specific source of *S. aureus* contamination could not be established in the present study. Nevertheless, the occurrence of enterotoxin-gene-positive *S. aureus* in food of animal origin represents a potential public health concern because staphylococcal enterotoxins are well-established causes of foodborne intoxication.

### 3.3. Prevalence of MRSA Isolates from Chicken Meat Samples

The prevalence of MRSA had been determined in 41.67% (40/96) of the recovered isolates, showing resistance to oxacillin and harbored *mecA* gene. Globally, the prevalence of MRSA in chicken meat is estimated to be around 5%, where the prevalence varies across different studies. High prevalence, as our findings show, have been reported previously in Bangladesh, where it was recorded in 54.9% and 37.10% [28]. Lower prevalence of MRSA was recorded in chicken meat (7.14%) in Tulsa, Oklahoma [29], and in China (8.1%) [3], Hong Kong (7%) [39], and Canada (1.3%) [38]. In Egypt, comparable MRSA prevalence was recovered in 44% (11/25), 44% (11/25), 52% (13/25), 40% (10/25), and 24% (6/25) of freshly slaughtered whole chicken carcasses, chicken burgers, chicken portions, chicken luncheon, and chicken sausages, respectively [29]. Conversely, it was detected in 5.6% of *S. aureus* isolated from chicken meat in a different Egyptian study [40].

### 3.4. Prevalence of VRSA Isolates from Chicken Meat Samples

In the current study, the *vanA* gene recovered from all *S. aureus* isolates showed resistance to vancomycin resistance by a prevalence of 5.20%. Previously, the prevalence of vancomycin resistance was 26.7% of *S. aureus* recovered from RTE shawarma and beef burger sandwiches in Zagazig Town, Egypt [41]. A substantially higher vancomycin resistance rate (80%) was documented among *S. aureus* isolates recovered from meat samples in Pakistan [42]. Similarly, a study conducted in southwestern Nigeria reported vancomycin resistance in all 138 *S. aureus* isolates obtained from ready-to-eat (RTE) foods in the Osogbo metropolis [43]. The detection of vancomycin resistance in 5.2% of the isolates is particularly alarming. When compared to earlier regional data, such as the study by Osman et al. [24], which reported lower rates of glycopeptide resistance in Egyptian poultry, our findings suggest a worrying upward trend in vancomycin-resistant *S. aureus* (VRSA) within the Nile Delta. This shift underscores the intensifying selection pressure caused by the unregulated use of avoparcin or similar glycopeptides in livestock production.

### 3.5. Antibiogram of S. aureus Isolates from Chicken Meat Samples

**Figure 2:**
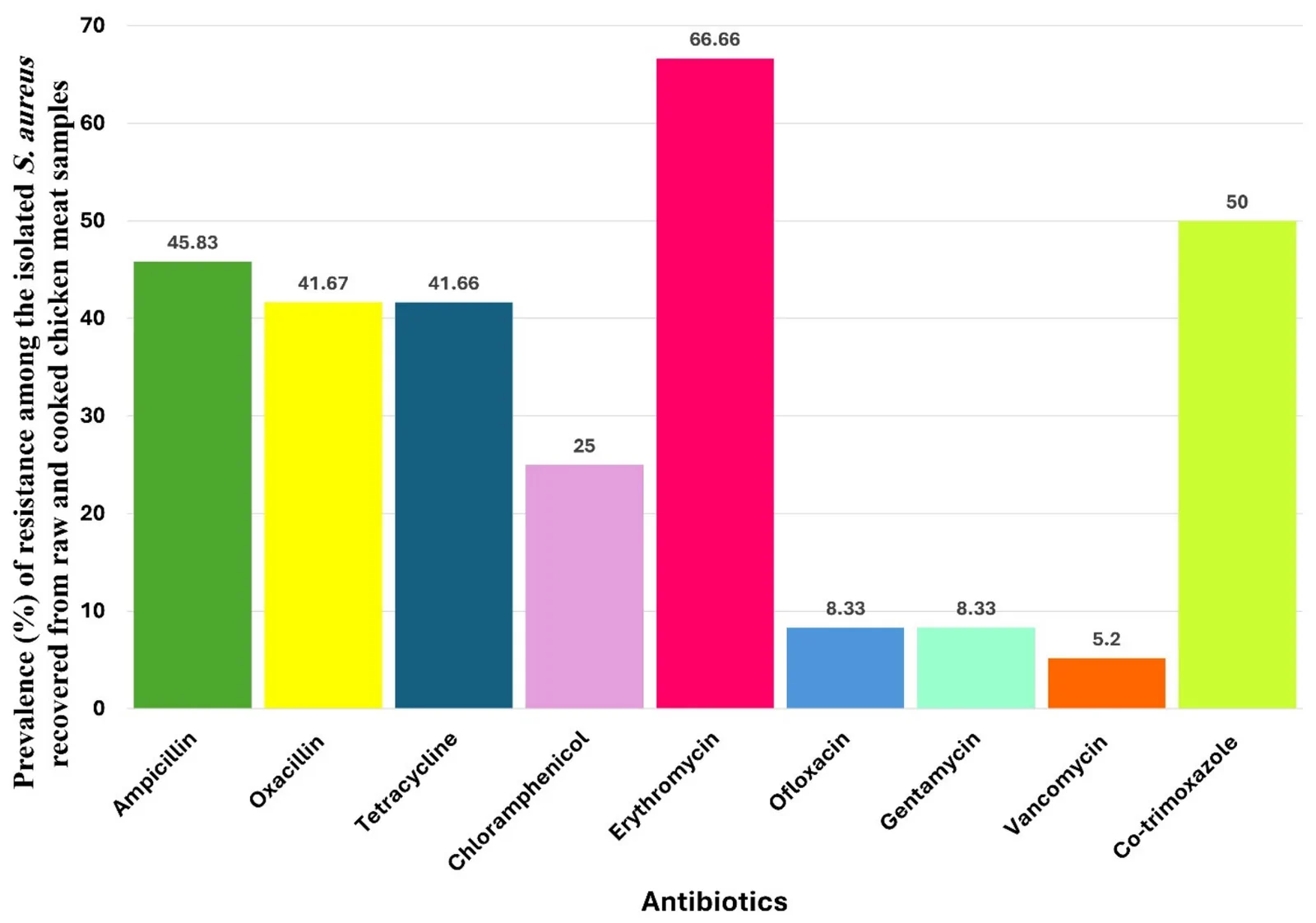
The prevalence of resistance against the different antibiotics tested among the *S. aureus* isolated from raw and cooked chicken meat samples, n = 96.

**Figure 3:**
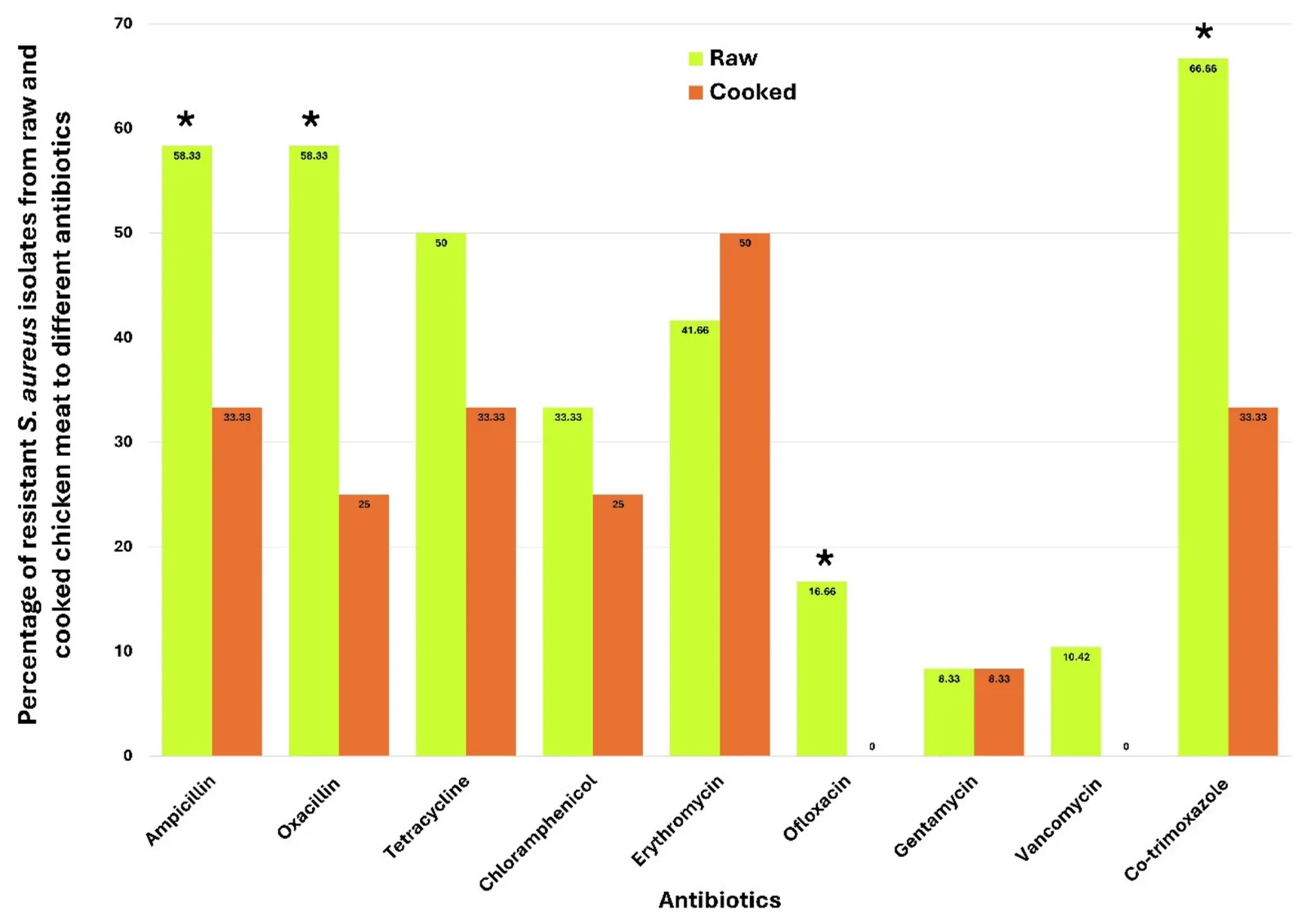
Prevalence of antibiotic resistance among *S. aureus* isolates recovered from raw and cooked chicken meat samples. Antibiotic resistance profiles of *S. aureus* derived from raw and cooked chicken meat samples (n = 96) in Egypt: ampicillin (AMP; 10 μg), oxacillin (OXA; 5 μg), ofloxacin (OFL; 5 μg), tetracycline (TET; 30 μg), chloramphenicol (C; 30 μg), gentamicin (GM; 10 μg), erythromycin (E; 15 μg), and trimethoprim/sulfamethoxazole (co-trimoxazole) (Co-T; 1.25/23.75 µg). *S. aureus* isolates were evaluated as resistant, intermediate, or susceptible according to CLSI [16,17]. The susceptibility against vancomycin (VAN) was tested by the broth microdilution method according to the CLSI using ranges of concentrations of 0.5–32 μg/mL. (*): significant at *p*-value < 0.05.

The antibiotic susceptibility of the confirmed *S. aureus* isolates in Figure 2 in the current study showed high resistance to erythromycin and cotrimoxazole at rates of 66.66% and 50%, respectively. In total, 50% of the *S. aureus* isolates were resistant to co-trimoxazole. Lower resistances were detected against vancomycin, gentamycin, and ofloxacin, at rates of 5.2%, 8.33%, and 8.33%, respectively. The resistance against ampicillin, oxacillin, tetracycline, and chloramphenicol was detected in 45.83%, 41.67%, 41.67%, and 25% of the *S. aureus* isolates, respectively. Resistance patterns of *S. aureus* isolates recovered from chicken meat from different countries to the corresponding antibiotics had been reported previously [31,44]. The antibiotic resistance profiles of *S. aureus* isolates showed a significant reduction in cooked meat compared to raw samples (n = 48 each). The resistance difference between raw and cooked chicken meat was significant (*p* < 0.05) for ampicillin, oxacillin, ofloxacin, and co-trimoxazole. Conversely, no statistically significant differences (*p* > 0.05) were observed for tetracycline, chloramphenicol, gentamycin, and erythromycin (Figure 3). This difference may be attributed to the greater reduction in viable resistant *S. aureus* following cooking.

In Egypt, penicillins and tetracyclines are widely used for disease treatment and non-therapeutic purposes, such as growth promotion and prophylaxis, in the poultry industry sector with no regulation [45]. The irrational usage of antimicrobials leads to MDR acquisition in many pathogenic food poisoning bacteria, such as *L. monocytogenes*, *S. aureus*, and other potentially pathogenic *Enterobacteriaceae*, such as *E. coli* and *K. pneumoniae*, recovered from food of animal origin [1,46,47,48,49]. Therefore, continuous monitoring of the antibiotic susceptibility profile of foodborne pathogens is imperative.

### 3.6. Antibiotic Resistance Profiles and Categorization of S. aureus Isolates

In the present study, in total, MDR were detected in 66.67% of the isolates recovered from raw and cooked chicken samples. Such a high prevalence was in accordance with the previous study, where MDR *S. aureus* recovered 87.5% of the samples [50]. On the other hand, many studies have revealed varying levels of MDR *S. aureus* prevalence in chicken meat samples, such as Bangladesh, where 100% of the *S. aureus* isolates were MDR [28]. In contrast, a lower prevalence of MDR *S. aureus* was detected in 26% (6/23) in the US [26]. Much lower MDR *S. aureus* prevalences recovered from food of animal origin were recorded as 1.0% (5/492) and 9.5% (4/42) in Taiwan and Northern Kazakhstan [51,52]. These findings feature the significance of monitoring and addressing the prevalence of multidrug-resistant *S. aureus*, including MRSA, in chicken meat to mitigate potential public health risks.

The prevalence of MDR *S. aureus* was significantly (*p* < 0.05) higher in raw chicken meat (83.33%, 40/48) compared to cooked samples (50.00%, 24/48). Although thorough cooking typically diminishes bacterial loads, samples in this cross-sectional study were gathered independently rather than as paired specimens. Consequently, the observed difference illustrates broader variations across retail samples. In addition to the thermal reduction in individual meat cuts during cooking, raw chicken may contain MDR *S. aureus* acquired during production, processing, handling, or cross-contamination, whereas adequate cooking temperatures can substantially reduce or eliminate viable *S. aureus*, including MDR strains, resulting in fewer resistant isolates being recovered from cooked samples. Thus, the lower MDR prevalence in cooked meat likely reflects the effect of heat treatment on bacterial survival rather than a direct loss of antimicrobial-resistance determinants.

### 3.7. Biofilm Formation Capability of S. aureus

**Figure 4:**
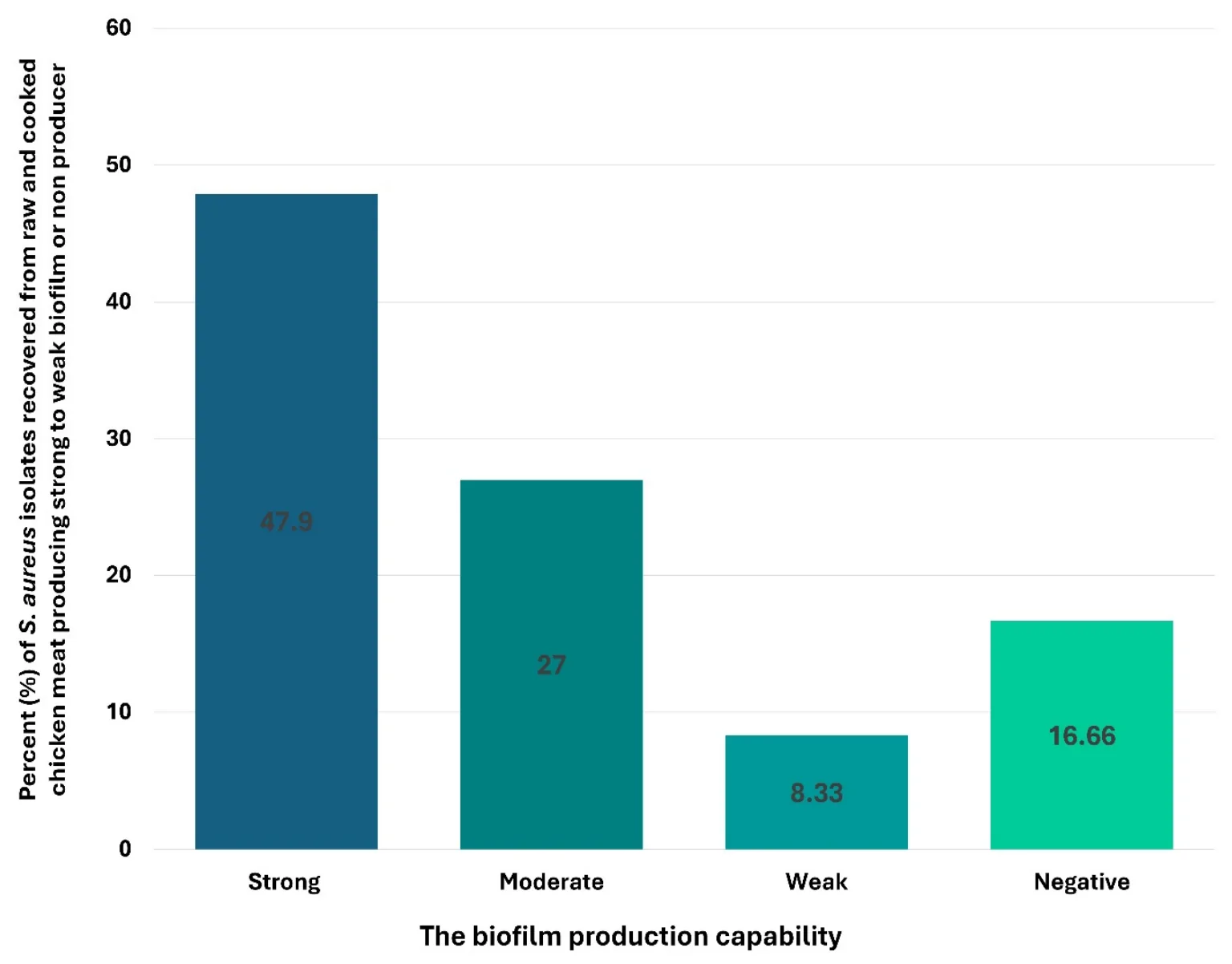
Prevalence of the biofilm capability formation among *S. aureus* that is isolated from raw and cooked chicken meat samples (n = 96). The samples are classified as strong, moderate, and weak biofilm producers, with some isolates producing no biofilm.

Biofilm production among the *S. aureus* isolates recovered in the current study was classified as strong (47.9%, 46/96), moderate (27%, 26/96), and weak (8.33%, 8/96), respectively, with 16.66% (16/96) of the isolates not producing biofilm at all (Figure 4). In total, 83.33% (80/96) of the isolates were biofilm producers. Similar findings were reported in an earlier investigation, in which *S. aureus* isolates collected from food-contact surfaces in dairy, meat-processing, and seafood-handling settings demonstrated biofilm-forming capacity. Other research has also highlighted *S. aureus*’s ability to form biofilms in food. For instance, studies showed that 89% (89 out of 100) of *S. aureus* isolates from various food sources in Bangladesh [53], and 61% (33 out of 54) from ready-to-eat foods in China [54], were biofilm producers.

**Figure 5:**
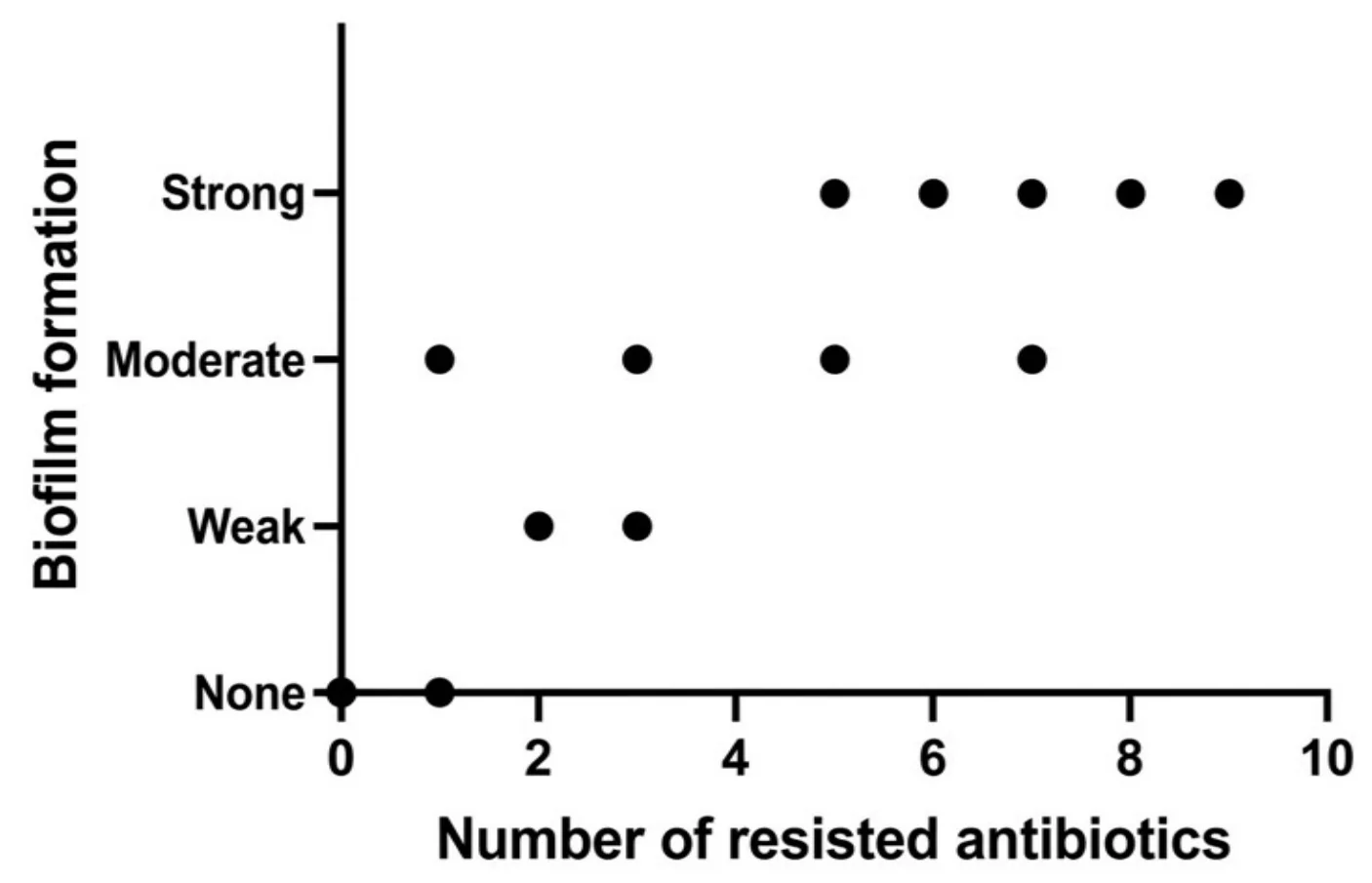
The correlation between antibiotic resistance and biofilm formation among *S. aureus* isolates recovered from raw and cooked chicken meat. The scatter plot illustrates the relationship between the number of antimicrobial agents to which an isolate exhibited resistance (*x*-axis) and the qualitative strength of biofilm production (*y*-axis: None, Weak, Moderate, and Strong).

There was a magnitude correlation between the antibiotic resistance profile of the *S. aureus* isolates and the biofilm formation capability *p*-value (˂0.0001) (Figure 5). The correlation between the capability of *S. aureus* to yield biofilm and increase its resistance to many antibiotics and become MDR has been confirmed in many studies previously [55,56].

### 3.8. Risk Assessment of Consumption of Chicken Meat Contaminated by S. aureus

The probability of developing illness following consumption of *S. aureus*-contaminated chicken meat samples examined in this study was estimated at 1.58 × 10^−4^ and 5.63 × 10^−5^ in raw and cooked chicken samples, respectively. However, these values are low when compared to the probability of illness value detected for *L. monocytogenes* in chicken meat in Egypt previously [44]; still, the risk from the consumption of *S. aureus* contaminated chicken meat is of concern, especially when the character of such isolates of multidrugs resistant to the most powerful antimicrobials used for the *S. aureus* infections treatment as oxacillin and vancomycin and of strong biofilm production, making them more resistant to treatment. While our quantitative model evaluates baseline exposure from raw chicken consumption as a worst-case scenario, actual foodborne risks in domestic settings are frequently exacerbated by cross-contamination of ready-to-eat surfaces, improper temperature abuse during storage, and post-cooking recontamination.

This study underscores the critical need for comprehensive risk assessments in the poultry industry, particularly regarding *S. aureus* contamination. By characterizing the antibiotic resistance profiles, virulence factors, and biofilm-forming abilities of *S. aureus* isolates from raw and cooked chicken meat, we highlight the potential public health risks associated with the consumption of contaminated poultry products. To further strengthen food safety strategies, future research should focus on quantifying the risks throughout the entire poultry production chain, from stable to table, in Egypt.

To mitigate this growing public health threat, a multi-tiered “One Health” strategy is required. At the farm level, the implementation of stringent “Antibiotic Stewardship Programs” is essential to transition away from the non-therapeutic use of critically important antimicrobials for growth promotion. At the retail level, regulatory bodies must ensure the strict implementation of Hazard Analysis and Critical Control Points (HACCP) protocols, supplemented by routine environmental swabbing of food-contact surfaces to identify reservoirs of biofilm-forming strains. Finally, at the consumer level, targeted public health campaigns are necessary to educate the public on the “Danger Zone” temperatures and the prevention of cross-contamination in domestic kitchens. Such integrated measures are vital to curbing the dissemination of MDR-MRSA from the poultry chain to the human population.

Study Limitations

A notable limitation of this study is that raw and cooked samples were collected independently in a cross-sectional design rather than as paired samples from identical meat before and after cooking. Consequently, differences in microbiological profiles between raw and cooked items reflect general retail conditions and thermal reduction effects rather than direct causal tracking of the cooking process. Furthermore, because exposure parameters and microbial concentrations were derived solely from Mansoura, these QMRA probabilities reflect local retail conditions and should not be broadly generalized. While this study provides valuable data on MRSA resistance to vancomycin in local markets, it is limited by its geographical focus on a single city. Furthermore, while we detected the MRSA and VRSA phenotypically and molecularly by screening the resistance genes, whole-genome sequencing (WGS) would be required in future studies to fully map the genetic lineage of these highly resistant strains.

## 4. Conclusions

This study confirms that retail chicken in Mansoura is a significant reservoir for MDR isolates, with 66.67% of total isolates exhibiting MDR, alongside specific occurrences of MRSA (41.67%) and VRSA (5.2%). Moreover, 83.33% of the isolates were capable of forming biofilms, a virulent factor linked to persistent infections and increased antibiotic resistance. The strong correlation observed between biofilm formation and antibiotic resistance suggests an association that may be linked to the enhanced resilience of these strains in the food chain. The calculated probability of illness from consuming contaminated chicken meat as estimated exposure probability, though numerically low, underscores the potential public health hazard, particularly when considering the high prevalence of resistant strains. Although limited to a single-city, cross-sectional design in Mansoura, these findings highlight the localized need for enhanced hygiene practices, thorough cooking guidelines, and prudent antimicrobial stewardship in regional poultry production. Our findings highlight the importance of shifting toward integrated surveillance (“One Health” approach) and stricter regulatory oversight of antibiotic use in Egyptian poultry production to safeguard public health.

## Data Availability

The original contributions presented in this study are included in the article. Further inquiries can be directed to the corresponding author.

## Abbreviations

The following abbreviations are used in this manuscript:

Abbreviation	Full term
AMR	Antimicrobial resistance
CFU	Colony-forming units
MDR	Multidrug-resistant
MRSA	Methicillin-resistant *Staphylococcus aureus*
VRSA	Vancomycin-resistant *Staphylococcus aureus*
MIC	Minimum inhibitory concentration
PCR	Polymerase chain reaction
DNA	Deoxyribonucleic acid
vanA	Vancomycin resistance gene *vanA*
Sea	Staphylococcal enterotoxin A gene
Seb	Staphylococcal enterotoxin B gene
